# Retraction: BRMS1 Suppresses Glioma Progression by Regulating Invasion, Migration and Adhesion of Glioma Cells

**DOI:** 10.1371/journal.pone.0270174

**Published:** 2022-06-14

**Authors:** The PLOS ONE Editors

Following the publication of this article [1], concerns were raised regarding the results presented in multiple figures. Specifically,

- In Fig 3A, the U251-BRMS1 24h panel appears to contain repetitive elements.
- The following results appear to partially overlap despite representing different experimental conditions:
  ○ The U251-BRMS1 panel in Fig 2D and the U251-BRMS1 panel in Fig 3C.
  ○ The U87-BRMS1 panel in Fig 2E and the U87-BRMS1 panel in Fig 3D.
  ○ The U251-Ctrl 0h panel and U251-BRMS1 0h panel in Fig 3A.

The corresponding author provided a PDF file containing images described as the original data underlying some of the results presented in this article, and they confirmed that the original files for the raw image data are no longer available.

The corresponding author stated that they do not know how the repetition in the U251-BRMS1 24h panel in Fig 3A was generated, and that they did not cut the image during assembly. The repetitive elements are also present in the image provided as original data, and this issue remains unresolved.

The corresponding author acknowledged that overlapping panels were included in error in Figs 2D, 2E and 3A, and they provided replacement panels. The replacement U251-BRMS1 panel for Fig 2D appears to partially overlap with the U251-BRMS1 panel in Fig 3C. Additionally, the images described as original data appear to match the published panels, and therefore, the partially overlapping regions remain.

In light of the unresolved concerns affecting multiple figure panels that question the integrity and reliability of these data, the *PLOS ONE* Editors retract this article.

YF and PM agreed with the retraction and apologize for the issues with the published article. All other authors either did not respond directly or could not be reached.
